# Perception, Awareness, and Practices Related to Burn First Aid Among the General Population in Qassim Region, Saudi Arabia

**DOI:** 10.7759/cureus.45879

**Published:** 2023-09-24

**Authors:** Khalid M Alkhalifah, Faisal Almutairi, Nouf S Almohaimeed, Lulwah S Alhumaidan, Lamees Alsulaim

**Affiliations:** 1 Department of Medicine, Unaizah College of Medicine and Medical Sciences, Qassim University, Unaizah, SAU; 2 Department of Surgery, Unaizah College of Medicine and Medical Sciences, Qassim University, Unaizah, SAU

**Keywords:** emergency, general public awareness, precipitation, burn first aid, burn patient

## Abstract

Objectives: This study aims to assess the perception, awareness, and practices related to burn first aid among the general population in the Qassim region of Saudi Arabia.

Methods: This is an observational, cross-sectional study that assesses perception, awareness, and practices related to burn first aid among the general population in the Qassim region of Saudi Arabia. The data was collected using questionnaires. The data was initially filtered and checked for completeness to rectify any errors or discrepancies. The Statistical Package for the Social Sciences (SPSS) version 24.0 (IBM SPSS Statistics, Armonk, NY, USA) was used for data analysis. The data was coded before entry into the software program. Descriptive statistics were applied, summarizing the data in terms of frequency and percentage. Chi-square tests were used for analyzing categorical variables and to determine the association between the groups, with significance set at a P-value of 0.05.

Results: Of the participants, 72.8% had previous knowledge regarding burns’ first aid management. Furthermore, 3% obtained knowledge and information about burn first aid from a certified course, while 21.1% obtained the information from the Internet. Of the respondents, 77.8% indicated that during the exposure to burn, they would remove accessories and clothes that covered the injured area. Of them, 79.4% noted that they would apply water to the injured area in case of exposure to burns. In addition, 61.9% of the respondents used honey as a home remedy to treat burns, and 30.1% used toothpaste to treat burns.

Conclusion: Of the general population in the Qassim region of Saudi Arabia, 72.8% had basic knowledge regarding burns’ first aid management. The study found certified courses and the Internet to be the main sources of information and knowledge about burns’ first aid management. The study found that clothes and accessories that covered the injured area should be removed when exposed to burns. In addition, cold water should be applied for a period of at least 10 minutes. The study found honey and toothpaste to be the most common home remedies used to treat burns. The use of pure honey is an accepted intervention in the treatment of burns due to its benefit in stimulating the rapid regeneration of tissues and decreasing incidences of scar formation. However, there are wrong beliefs about the use of toothpaste in cases of burns because it exacerbates the initial injury, making it even worse. There are significant differences in the perception, awareness, and practice of the general population according to their education level (P-value = 0.003) and employment (P-value = 0.007).

## Introduction

Injuries from burns are underestimated and can happen to any individual at any moment, anywhere. Although most burn injuries originate and are brought on by heat from hot liquids, solids, or flames, the burns can also be brought on by abrasion, freezing, radiation, chemical, or electric sources [1]. Moreover, injuries related to burns are one of the most important issues affecting the general population. According to World Health Organization reports, the estimated mortality caused by burn injuries is 180,000 per year, which is the fourth most prevalent form of trauma globally [2]. In Saudi Arabia, the annual incidence of burn-related injuries varied from 112 to 518 per 100,000, with householders representing 72%-94% of causes [3]. In addition, it is considered one of the most costly and harmful public health crises due to its significant physical, functional, and mental effects [4,5].

First aid is the rapid giving of basic initial medical care to a person experiencing an unexpected trauma or health issue. Moreover, effective initial treatment is simple to administer, relieves pain, minimizes the impact of the damage, and beneficially impacts further professional therapy [6]. In addition, the importance of first aid for burns includes more than one aspect, including the psychological and aesthetic aspects, and the most important aspect is that if it is done early, regardless of specialized treatment, it reduces future complications, unlike if it is delayed [7]. Therefore, each member has to increase their understanding of first aid and basic burn treatment practices [8]. However, each kind of burn requires different first aid procedures. For example, rolling on the floor and then applying cool running water to a flame burn will significantly decrease morbidity [2].

Many people perform first aid incorrectly. The use of natural therapies that are not validated by science is one of these wrong interventions. For instance, ice may cause vasoconstriction, which makes tissue breakdown worse. Actually, a lot of individuals employ traditional treatments such as toothpaste, eggs, soil, mayonnaise, mustard, butter, lavender oil, and a lot more, which are not helpful and may even be dangerous [9].

Due to the importance of first aid burn-related injuries to public health and their long-term consequences, and with a lack of data in the Qassim region, our aim in this study is to evaluate perception, awareness, and practices related to burn first aid among the general population in the Qassim region of Saudi Arabia.

## Materials and methods

Study design, duration, and setting

This was an observational, cross-sectional study. The questionnaire was distributed from June 20, 2023, to August 20, 2023. An online questionnaire was distributed through social media applications (WhatsApp, Twitter, and Facebook) to invite participants from the Qassim region, Saudi Arabia, to participate in the study.

Sample size and sampling technique

The sample size was calculated using Epi Info software version 7.2.2.6 (Centers for Disease Control and Prevention, Atlanta, GA, USA), with a 99% confidence interval, 80% power, and 50% expected frequency. The estimated sample size was 663. Participants were recruited through a convenient sampling technique and were requested to provide informed consent. The study’s objectives were presented to the participants, and informed consent was obtained. Data were collected from all participants who met the following criteria: Qassim citizens, aged 18 years or above, and agreed to participate.

Data collection methods

A validated questionnaire, previously used in another study [9], was employed. The questionnaire was adapted and expanded to align with the study’s objectives. It consisted of three parts. The first part gathered biographical data and information about previous exposure and knowledge of burns. The second part included 16 clinical scenarios to assess participants’ knowledge and perceptions regarding first aid management of burns. The last part consisted of nine questions to assess burn-related nutrition practices.

Data management and analysis plan

The data were initially cleaned and checked for completeness to rectify any errors or discrepancies. The Statistical Package for the Social Sciences (SPSS) version 24.0 (IBM SPSS Statistics, Armonk, NY, USA) was used for data analysis. The data were coded before entry into the software program. Descriptive statistics were applied, summarizing the data in terms of frequency and percentage. Chi-square tests were used for analyzing categorical variables and to determine the association between the groups, with significance set at a P-value of 0.05.

Ethical considerations

Ethical approval was obtained from the Research Ethics Committee of Qassim University (23-54-03). Electronic consent was obtained from each participant before conducting the study. All collected data was kept confidential and used solely for research purposes.

## Results

Table 1 presents the social characteristics of the participants. Regarding age, the results showed that the age of most of the participants ranged between 19 and 25 years, representing 44.3%, while only 2.3% were 46 years old or older. In terms of gender, 58.7% of the participants were female, and 41.3% were male. The results of the study showed that more than half of the participants (69.8%) had an undergraduate degree and that 15.6% of them were employed as healthcare providers. The vast majority of the participants (98.6%) were Saudi nationals, while only 1.4% of them were non-Saudi nationals. The results showed that 32.3% of the participants were students, 20.6% were teachers, and 17.6% were unemployed. In terms of monthly income, the findings noted that half of the participants (50.1%) earned between 10,000 and 20,000 Saudi Riyals monthly. The vast majority (79.7%) of the respondents indicated that they were living with children or adolescent teenagers (under 18 years), while 20.3% were not living with children under 18 years. The findings revealed that the majority of the participants (21.6%) resided in the Buraydah region. Of them, 18.3% resided in Riyadh Al Khabra, 17.3% resided in Bukayriyah, and only 1.9% resided in other regions.

**Table 1:** Social characteristics of the participants (N = 833)

Social characteristics	Frequency and proportion (number (%))
Age (years)	
15-18	147 (17.7%)
19-25	369 (44.3%)
26-35	134 (16.1%)
36-45	163 (19.6%)
46 and more	19 (2.3%)
Gender	
Male	344 (41.3%)
Female	489 (58.7%)
Level of education	
Illiterate	10 (1.2%)
Primary	23 (2.8%)
Middle	54 (6.5%)
High school	139 (16.7%)
Undergraduate (bachelor’s degree)	581 (69.8%)
Master, PhD	35 (4.2%)
Nationality	
Saudi	821 (98.6%)
Non-Saudi	12 (1.4%)
Employment	
Unemployed	147 (17.6%)
Student	269 (32.3%)
Teacher	172 (20.6%)
Office	95 (11.4%)
Healthcare provider	130 (15.6%)
Others	21 (2.5%)
Monthly income	
Less than 10,000 Saudi Riyals	137 (16.4%)
Between 10,000 and 20,000 Saudi Riyals	417 (50.1%)
Between 21,000 and 30,000 Saudi Riyals	156 (18.7%)
More than 30,000 Saudi Riyals	123 (14.8%)
Living with children, adolescents teenagers (under 18 years)	
Yes	664 (79.7%)
No	169 (20.3%)
Residence	
Buraydah	180 (21.6%)
Bukayriyah	144 (17.3%)
Almudhanab	67 (8.1%)
Alrass	105 (12.6%)
Albadayie	81 (9.7%)
Unayzah	87 (10.5%)
Riyadh Al Khabra	152 (18.3%)
Others	16 (1.9%)

The results in Table 2 show that 72.8% of the participants had previous knowledge regarding burns’ first aid management, while 27.2% of them did not have the information. Of the participants, 70.1% had a history of self-exposure to burn injuries, 25.6% of them did not, and 4.3% did not remember. Furthermore, 77.3% of the participants indicated that they had a history of family members being exposed to burn injuries, 21.6% had no history, and 1.1% did not remember. Of the participants, 67.9% had given first aid to a burn victim, while 32.1% had never done so.

**Table 2:** Knowledge and awareness of burns’ first aid management

Questions	Yes (frequency/percentage)	No (frequency/percentage)	I don’t remember (frequency/percentage)
Have you ever received information about burn’s first aid management?	606 (72.8%)	227 (27.2%)	-
Is there a history of self-exposure to burn injury?	584 (70.1%)	213 (25.6%)	36 (4.3%)
Is there a history of a family member being exposed to burn injury?	644 (77.3%)	180 (21.6%)	9 (1.1%)
Have you ever done first aid management for a burn victim?	566 (67.9%)	267 (32.1%)	-

In terms of sources of information for burn first aid management (Table 3), 23% of the participants obtained knowledge and information about burn first aid from a certified course, 21.1% from the Internet, and 11.3% from medical brochures and pamphlets. Exactly 8.9% of the respondents had obtained the information from social media applications, while 7.7% from the curriculum of college. A smaller proportion (1.2%) obtained the information from newspapers and during a hospital visit (1.3%).

**Table 3:** Sources of information for burns’ first aid management

Source of information	Frequency	Percentage
Curriculum of school	57	6.9%
Curriculum of college	64	7.7%
Medical brochures and pamphlets	94	11.3%
Newspaper	10	1.2%
Television/radio	13	1.6%
Internet	176	21.1%
Certified courses	192	23%
Social media applications	74	8.9%
While visiting hospital	11	1.3%
Text messages	10	1.2%
Other	11	1.3%
I have not learned about burns first aid management	46	5.5%

The results in Table 4 show that 77.8% of the respondents indicated that during exposure to burn, they would remove accessories and clothes that covered the injured area. Of them, 79.4% noted that they would apply water to the injured area in case of exposure to burns. Additionally, 53.1% of the respondents thought that if someone catches fire and is in flames, they should not wrap the person in thick material, such as a wool or cotton coat, rug, or blanket. In addition, 71.3% believed that they would remove a child’s clothes and then pour water on their body in case hot, boiling oil spilled on a child’s chest in the kitchen. Of the respondents, 65.1% thought that they would stop moving, lie down, and then roll over the ground if their clothes caught fire during a picnic, and 71.6% of them thought that they would put the affected area under cold water for 10-20 minutes if boiling water spilled on their hands during a social meeting.

**Table 4:** Dealing with burns

Questions	Yes (frequency/percentage)	No (frequency/percentage)	I don’t know (frequency/percentage)
During exposure to burns, I will remove accessories and clothes that cover the injured area.	648 (77.8%)	185 (22.2%)	-
During exposure to burns, I will apply water to the injured area.	661 (79.4%)	143 (17.2%)	28 (3.4%)
Always seek medical help if the age of the victim is ˂4 years or ˃60 years.	426 (51.2%)	358 (43%)	48 (5.8%)
Always seek medical help if the hands, feet, face, groin, buttocks, or a major joint is burnt.	446 (53.6%)	387 (46.4%)	-
Always seek medical help if it is a chemical or electrical burn.	540 (64.8%)	252 (30.2%)	42 (5%)
In case of burns, I will keep blowing/fanning the burn.	373 (44.8%)	336 (40.3%)	124 (14.9%)
If someone catches fire and is in flames, wrap the person in a thick material, such as a wool or cotton coat, rug, or blanket.	344 (41.4%)	442 (53.1%)	46 (5.5%)
In case of flame burns, I will remove the clothes stuck in the injured area.	627 (75.3%)	157 (18.9%)	48 (5.8%)
In case of chemical burns, I will remove the clothes stuck in the injured area.	601 (72.1%)	181 (21.7%)	52 (6.2%)
If there are large areas or very deep burns, give water/milk by mouth.	392 (47.1%)	291 (35%)	149 (17.9%)
Cover the affected areas with a clean cotton cloth after removing the surrounding dress.	553 (66.4%)	243 (29.2%)	37 (4.4%)
In case hot boiling oil spills on the chest of a child in the kitchen, I will remove the child’s clothes and then pour water on his body.	594 (71.3%)	180 (21.6%)	59 (7.1%)
If someone’s clothes catch fire during a picnic, I will stop moving, lie down, and then roll over the ground.	542 (65.1%)	236 (28.3%)	55 (6.6%)
During a social meeting, if boiling water spills on someone’s hand, I will put the affected area under cold water for 10-20 minutes.	596 (71.6%)	146 (17.5%)	91 (10.9%)
Do you think that home remedies that are mentioned above can be used for burned injured areas?	526 (63.1%)	217 (26.1%)	90 (10.8%)

Based on the results in Table 5, the vast majority of the participants (79.3%) believed that cold water should be applied in cases of burns. Of them, 15.1% thought it should be warm water, and 2.6% of the respondents thought hot water should be applied in case of a burn. The results showed that 31.8% of the respondents would apply water for 10-15 minutes, 24.1% believed that they would apply water on burns for more than 15 minutes, and 21.6% of them thought water should be put on burns for 5-10 minutes. Additionally, 14.2% of them thought that water should be applied to burns for less than five minutes. In terms of home remedies, 61.9% of the respondents used honey to treat burns, 30.1% used toothpaste to treat burns, and 4.1% used an ice pack.

**Table 5:** Participants’ opinions and response practices to various burn situations

Research question	Categories	Frequency	Percentage
Water temperatures to be applied on burn	Cold	661	79.3%
	Warm	126	15.1%
	Hot	21	2.6%
	I will not apply water	25	3%
Duration of applying water to a burn	Less than 5 minutes	118	14.2%
	Between 5 and 10 minutes	180	21.6%
	Between 10 and 15 minutes	265	31.8%
	More than 15 minutes	201	24.1%
	I don’t know	60	7.2%
	I will not apply water	9	1.1%
Home remedies used on burn	Honey	515	61.9%
	Toothpaste	251	30.1%
	Ice cold compress	34	4.1%
	Tomato paste	12	1.5%
	White flour	9	1.1%
	Milk or yogurt	11	1.3%
	I have never used natural remedies	-	-

The results of Table 6 show that there is a significant difference in the knowledge of the participants about first aid according to their education level (P-value = 0.003), and this difference is between those in the medical field and those in the academic field. Also, there is a significant difference in their knowledge according to their employment, where the P-value is 0.007, and the difference is between those in the medical field and those in the academic field. The results revealed significant differences in burn first aid practice by the study members according to their education level and their employment, where the P-value was 0.000.

**Table 6:** Differences in the knowledge of the participants about first aid regarding their education level and employment df: degrees of freedom

	Sum of squares	df	Mean square	F	Significance
Education level					
Between groups	1.381	5	0.276	1.369	0.002
Within groups	74.227	827	0.089		
Total	75.608	832			
Employment					
Between groups	3.164	5	0.633	3.263	0.007
Within groups	74.468	827	0.090		
Total	77.632	832			

## Discussion

The aim of this study is to evaluate the general population’s perception, awareness, and practices pertaining to burn first aid within the Qassim region in Saudi Arabia. The implementation of first aid measures is of paramount importance in enhancing the prognosis and mitigating the financial implications associated with burn injuries [10]. The findings of the study indicated that a majority of the participants, specifically (72.8%), possessed prior knowledge pertaining to the administration of burns’ first aid. Conversely, a minority of participants, accounting for 27.2%, reported having no previous experience in this area.

The results showed that 23% of the participants obtained knowledge and information about burn first aid from a certified course, 21.1% of them obtained the information from the Internet, and 11.3% of the participants obtained the information from medical brochures and pamphlets. The results of this study support the findings of Kattan et al. (2016) [4], which indicated that the largest proportion of participants in their study (44.4%) acquired knowledge of burn first aid from educational courses and online resources. This suggests that utilizing classes and the Internet could potentially serve as effective means to enhance public knowledge and consciousness regarding burn injuries.

Based on the findings, it was observed that a significant majority of the respondents (77.8%) held the belief that accessories and clothing covering the affected region ought to be eliminated when subjected to burn injuries. Most of the participants also expressed the belief that the use of water is necessary in the event of a burn injury. The results align with the findings of a previous study conducted in Saudi Arabia [11], which indicated that a significant proportion of participants were aware of the appropriate actions to take in the event of burns, such as removing clothing, applying water, and seeking medical attention. Based on the results, a significant proportion of the participants (79.3%) demonstrated awareness regarding the appropriate use of cold water in the event of a burn. Among these individuals, 31.8% specified that water should be applied for a duration ranging from 10 to 15 minutes, while 24.1% indicated that water should be applied for a period exceeding 15 minutes. The results align with the conclusions reported by Harvey et al. [12]. A research study conducted in Wales demonstrated that a significant majority of participants (82%) reported utilizing cold water as a regular practice for treating burn injuries. Furthermore, within the subset of individuals who employed cold water, a notable proportion of 9.4% were aware of the recommended length of time for this treatment, which is typically 10-20 minutes. The assertions are additionally corroborated by the research conducted by Scheven et al. [13]. A research investigation conducted in the province of KwaZulu-Natal revealed that 26% of individuals utilized cold water as a treatment for burns, while a mere 1% of these individuals possessed knowledge regarding the recommended duration of at least 10 minutes for which water should be applied.

The findings of the survey indicate that a significant proportion of the respondents (61.9%) employed honey as a domestic cure for burn treatment, while 30.1% of the participants reported using toothpaste for the same purpose. The results align with the data reported by Kattan et al. [4], which indicated that honey and toothpaste were the predominant herbal treatments employed for burn treatment, accounting for 69.9% and 53.7% of usage, respectively. A significant proportion (61.9%) of the research population employed honey as a first aid measure, a practice that has gained acceptance due to its associated advantages. Numerous studies have demonstrated that the application of pure honey on burns can effectively promote the expeditious regeneration of tissues and reduce the likelihood of scar formation [14,15]. The utilization rate of toothpaste among the population cannot be deemed as low, as it is an incorrect practice that has the potential to worsen the initial damage [16]. Therefore, it is imperative to enhance public knowledge concerning this erroneous traditional notion. Moreover, the findings indicated substantial disparities in perception, awareness, and behavior based on participants’ educational attainment and work status. These outcomes will assist the researcher in identifying certain segments of the community that would benefit from enhanced education regarding burn first aid.

Study limitation

Given that the study has an observational, cross-sectional design, only the relations between factors can be determined, not causalities.

## Conclusions

In conclusion, the majority of the general population in the Qassim region of Saudi Arabia had basic knowledge regarding burns’ first aid management. The findings emphasize the importance of public awareness and education in addressing burn injuries, with the majority of participants showing prior knowledge about first aid. The study suggests that educational courses and online resources can be effective tools for disseminating this crucial information. Additionally, the results highlight the need for improved awareness regarding the correct use of domestic remedies such as honey and the dangers associated with using toothpaste for burn treatment. Nevertheless, these results can inform targeted educational interventions to improve public awareness and behavior regarding burn injuries, ultimately contributing to better outcomes and reduced financial burdens associated with burns in the region.
